# Forensic psychiatric assessment process and outcome in state patients in KwaZulu-Natal, South Africa

**DOI:** 10.4102/sajpsychiatry.v24i0.1142

**Published:** 2018-03-20

**Authors:** Ahlem Houidi, Saeeda Paruk, Benn Sartorius

**Affiliations:** 1 Department of Psychiatry, University of KwaZulu-Natal, South Africa; 2 Public Health Medicine, School of Nursing and Public Health, University of KwaZulu-Natal, South Africa

## Abstract

**Background:**

Individuals who were charged with a serious offence may be referred by courts for forensic psychiatric assessment. The court may declare them as state patients if they are found unfit to stand trial or not criminally responsible because of mental illness or defect.

In forensic psychiatry practice, there may be challenges in the forensic psychiatric observation process, and discrepancies may occur between the clinician report and the court’s decision.

**Objectives:**

To describe elements of the forensic psychiatric observation and discuss the legal correlates associated with the admission of state patients.

**Method:**

A retrospective study of the forensic psychiatric observation records of 91 newly admitted state patients at a forensic unit in KwaZulu-Natal over a 3-year period.

**Results:**

A total of 71 state patients (78.02%) were found not fit to stand trial and 10 patients (10.99%) were not criminally responsible. Nine patients (9.89%) were fit to stand trial and criminally responsible but still declared state patients and 13 state patients (14.29%) did not commit a serious offence. There was correlation for diagnosis between the assessing and the treating psychiatrists.

**Conclusion:**

The findings of the forensic observation were not always considered by the courts. Individuals found fit to stand trial, those found criminally responsible and those who did not commit serious crimes were declared state patients.

A better understanding of legal dispositions for mentally ill offenders and an active collaboration between judicial and mental health systems may contribute in developing national guidelines for observation and admission of state patients.

## Introduction

Since at least the 17th century, the provision of fitness to stand trial has been recognised in many countries. It was established to protect the reliability and accuracy of a trial, the dignity of the criminal justice system and the effectiveness of punishment.^1^

In South Africa, accused may be referred from court to a psychiatric forensic unit for a forensic psychiatric observation in terms of section *79 of the Criminal Procedure Act* (CPA) No. 51 of 1977.^2^ The aim is to assess whether the accused has a mental illness or intellectual disability, the fitness to stand trial (section 77) and/or the criminal responsibility (section 78: ability to appreciate the wrongfulness of their actions, or to act in accordance with such an appreciation).^2^ The assessors submit the forensic psychiatric report to court after a period not exceeding 30 days. The judicial system makes decisions based on this report, but this is not binding to the court. Individuals who have committed serious offences and who are found unfit to stand trial and/or not criminally responsible may be referred by the court for admission to a forensic psychiatric unit as state patients under section 42 of the Mental Health Care Act (MHCA) for an indefinite period.^3^ The purpose of the admission as a state patient is not punishment but rather treatment, care and rehabilitation, while simultaneously monitoring and managing their potential risk to the community.^4^ When state patients become capable of understanding the court proceedings so as to make a proper defence, they may be prosecuted and tried.^2^

In the province of KwaZulu-Natal, only one psychiatric unit provides for the observation of accused referred from courts, and there are three admitting facilities for state patients. Generally, there is a limited number of forensic psychiatric facilities and a shortage of psychiatrists involved in forensic psychiatry in the state sector.^4^ This may have an impact on the standard of the forensic observation. Furthermore, at times, courts may make controversial decisions that may seem unreasonable; for example, after having been found fit and sound of mind, accused were sent from court to a forensic psychiatric unit for treatment.^5^ These are some of the challenges that face forensic psychiatry globally and particularly in poorly resourced settings. There is a paucity of South African studies on this aspect of forensic psychiatry practice.

This article was part of a larger study describing the demographic, clinical and forensic profile of state patients in KwaZulu-Natal.^5^ The article aimed to describe the process and discuss the finding and legal outcome of the forensic psychiatric assessment in 91 state patients admitted to a forensic unit in KwaZulu-Natal, South Africa. It also aimed at exploring the factors associated with the finding of the observation, as well as the correlation between the clinical assessment at admission as state patient and at observation.

## Method

### Study design and setting

This was a retrospective, descriptive study of clinical records of state patients admitted to forensic psychiatric hospital in KwaZulu-Natal from 01 June 2013 to 31 May 2016.

### Participants

There were 878 admissions for forensic psychiatric observation assessment over the same period. The study sample comprised 91 newly admitted male and female state patients aged between 15 and 65 years.

### Measurements

Data were collected from hospital charts, including 91 medical files and 91 observation files. The data sheet included socio-demographic (age, level of education, marital status, employment), clinical (DSM 5 diagnosis on admission as state patient, past psychiatric history), forensic (age at the time of the offence, type of offence, past forensic history, victim’s age, rape, attitude towards the crime) and observation (diagnosis in the observation report, finding in terms of sections 77 and 78 of the CPA, number of observations, period between crime and observation and between observation and admission) factors. A forensic psychiatrist extracted the data from the hospital records.

### Statistical analysis

Data were imported into Stata 13.0 (StataCorp. 2013. Stata Statistical Software: Release 13. College Station, TX: StataCorp LP) for processing and analysis. Categorical variables were summarised using frequency tables. Association between fit and/or unfit classification and demographic characteristics, diagnosis and type of crime/offence were assessed using the Fisher’s exact test given the small sample size. Agreement between primary diagnosis and diagnosis at observation was assessed using the Kappa statistic. A *p*-value of < 0.05 was considered statistically significant.

## Results

The sample population represented 10.36% of the total admissions for observation over the study period. Thirteen (14.29%) of the state patients did not commit a serious offence. The socio-demographic profile was predominantly single (*n* = 89, 97.80%), unemployed (*n* = 89, 97.80%), with intellectual disability (*n* = 33, 36.26%). Forensic factors were presented in another manuscript in press.

### Forensic psychiatric assessment process time

**FIGURE 1:**
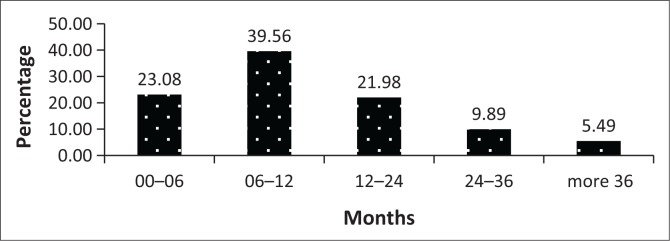
Period of time between crime and observation.

**FIGURE 2:**
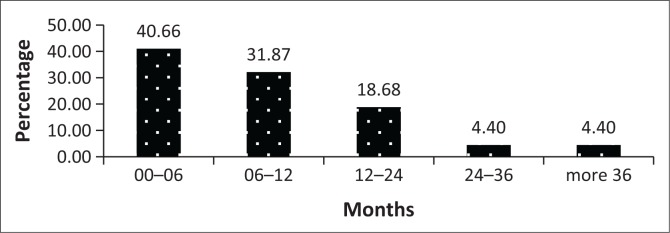
Period of time between observation and admission.

Prior to the admission as state patients, 21 (23.08%) accused were referred from court for observation within 6 months of the crime (Figure 1). Thirty-seven (40.66%) accused were admitted as state patients within 6 months after the psychiatric observation (Figure 2). Fifteen (16.48%) patients had more than one observation and nine (9.89%) had the second observation done in one year’s time.

### Forensic psychiatric observation finding

All patients were assessed in terms of both sections *77 and 78 of the CPA*. There were no comments on the ability to appreciate the wrongfulness of the act at the time of the crime (criminal responsibility) in 65 (71.43%) patients. A total of 71 (78.02%) state patients were found not fit to stand trial, 10 (10.99%) patients were unable to appreciate the wrongfulness of the act and 9 (9.89%) patients were considered both fit to stand trial and criminally responsible (Table 1).

**TABLE 1:** Finding of fitness to stand trial and criminal responsibility in state patients at psychiatric observation

Conclusion in the report	*N*	%
Fit and NCR	1	1.10
Fit and CR	9	9.89
Unfit and no comments	54	59.34
Unfit and CR	8	8.79
Unfit and NCR	9	9.89
Fit and no comments	10	10.99

**Total**	**91**	**100.00**

NCR, not criminally responsible; CR, criminally responsible.

### Clinical assessment at observation and at admission as state patient

At the time of the observation 34 (37.36%), patients were diagnosed with intellectual disability compared to 33 (36.26%) on admission as state patient, and 5 patients (5.49%) had no mental illness diagnosed according to the observation report. After admission as state patients, 43 (47.25%) state patients denied committing the crime and 19 (20.88%) expressed regret at committing the crime. There was a correlation between the diagnoses made at admission as state patient and during observation (*p* < 0.001) (Table 2).

**TABLE 2:** Correlation between diagnosis in observation report and diagnosis at admission.

Diagnosis at admission	Diagnosis in observation report			
	Intellectual disability	Schizophrenia	Other	Total
Intellectual disability	29	0	3	32
Schizophrenia	2	10	14	26
Other	3	3	27	33

**Total**	**34**	**13**	**44**	**91**

### Analysis of association between the profile of state patients and the finding of fit and not fit to stand trial

There was an association between the single status and unemployment status and not fit to stand trial (Table 3).

**TABLE 3:** Association between fit/unfit classification and demographic characteristics, diagnosis and type of crime and/or offence.

Characteristic	Unfit to stand trial (*N* = 71) *N* (%)	Fit to stand trial (*N* = 20) *N* (%)	*p* (i)
**Level of education**			
No education	3 (75.0)	1 (25.0)	0.171
High school	22 (68.8)	10 (31.3)	-
Primary school	46 (83.6)	9 (16.4)	-
**Marital status**			
Single	71 (79.8)	18 (20.2)	0.046
Married	0 (0.0)	1 (100.0)	-
Widowed	0 (0.0)	1 (100.0)	-
**Employment**			
Not working	71 (79.8)	18 (20.2)	0.046
Working	0 (0.0)	2 (100.0)	
**Age at the time of the crime (years)**			
15–25	31 (79.5)	8 (20.5)	0.964
26–35	25 (75.8)	8 (24.2)	-
36–45	9 (75.0)	3 (25.0)	-
46–55	5 (83.3)	1 (16.7)	-
56–65	1 (100.0)	0 (0.0)	-
**Past psychiatric history**			
No	43 (84.3)	8 (15.7)	0.102
Yes	28 (70.0)	12 (30.0)	-
**Past forensic history**			
No	52 (80.0)	13 (20.0)	0.414
Yes	18 (72.0)	7 (28.0)	-
**Rape offence**			
No	23 (69.7)	10 (30.3)	0.148
Yes	48 (82.8)	10 (17.2)	-
**Type of crime**			
Offence against persons	62 (77.5)	18 (22.5)	0.876
Offence against property	6 (85.7)	1 (14.3)	-
Both	2 (66.7)	1 (33.3)	-
Other	1 (100.0)	0 (0.0)	-
**Age of victim**			
Child	41 (82.0)	9 (18.0)	0.285
Adult	23 (69.7)	10 (30.3)	-
**Diagnosis at admission**			
Intellectual disability	26 (81.3)	6 (18.8)	0.39
Schizophrenia	22 (84.6)	4 (15.4)	-
Other	23 (69.7)	10 (30.3)	-
**Diagnosis at observation**			
Intellectual disability	30 (88.2)	4 (11.8)	0.169
Schizophrenia	10 (76.9)	3 (23.1)	-
Other	31 (70.5)	13 (29.5)	-

i: Pearson chi-squared test for categorical covariate (or Fisher’s exact test if cell count < 5).

## Ethical consideration

Ethical approval was obtained from the University of KwaZulu-Natal Bioresearch Ethics Committee (BE 190/16), and permission was obtained from the participating hospital and the Department of Health.

## Discussion

The key findings of this study were the long delay between the time of the offence and the observation period. Also, the psychiatric observation report findings were not always considered by the courts in determining patient detention. Additional concerning findings were that a proportion of cases were declared state patients without committing a serious offence or despite being fit to stand trial and criminally responsible. These findings suggest that the accused’s rights to a fair observation, trial and outcome may have been compromised and raise ethical issues.

Furthermore, there was an association between unfitness to stand trial and being single or unemployed. This finding is expected as the demographic profile of the study population is predominantly single and unemployed with majority found not fit to stand trial.^5^

The finding that approximately 1 out of every 10 observation cases was admitted as a state patient back to the unit should be considered with caution. Some state patients may have been admitted to another forensic psychiatric unit in the province based on their residential address. Other decisions may also have been made by the court (involuntary admission, conditional or unconditional release, imprisonment).^2^ Other situations may include abscondment of the accused, missing dockets or delays in the admission process. Accused were detained in the legal system and sent from court for observation often more than 6 months after the crime, and one-third were sent more than a year later. This delay in referrals (up to 3 years) for observations appears to be a local challenge as Yap et al. reported that 70.6% of the offenders under criminal commitment in Singapore were referred for observation within 1 week after the crime.^6^ This delay is a concern as the mental state of the offender may be altered and his or her recall ability impaired. In addition, an injustice may occur as an offender may have to be detained longer than necessary.^7^ In the South African context, delays may be because of the court proceeding limitations or the limited number of forensic psychiatric facilities and a shortage of qualified psychiatrists involved in forensic psychiatry in the state sector.^4^ Other factors include delays in transporting accused from prison to hospital, delays in getting accused arrested, abscondment of accused while they are out on bail, delays by prosecutor to book the bed and long postponements between court dates. The long waiting list for observations is another contributing factor to delay in referrals. In the United States, many states have shifted to outpatient evaluations.^8^ In some countries, to avoid unnecessary remands in custody, court-based psychiatric diversion schemes have been established, enabling magistrates to obtain rapid psychiatric assessments. This also facilitates arrangements for voluntary or compulsory admission with or without discontinuation of the criminal proceedings or for remand in hospital for forensic mental assessment.^6^ There are ethical issues surrounding the processing time of the psychiatric observation related to these delays. Thus, there is a need to address these issues as accused may not benefit from a fair observation and subsequently from a fair trial. It would be recommended that instead of the preliminary assessment and the admission for assessments, which may also be a delaying factor, forensic psychiatric observations be conducted for all accused on an outpatient basis at psychiatric institutions nearest to their homes. The psychiatrist or a panel in case of serious offence may then recommend the in-patient 30-day observation when absolutely needed (if the accused comes from very far and there is a need for input from the multidisciplinary team). The main purpose is to prevent ethical issues related to delays and to avoid compromising the fairness of the assessment.

This study also found a delay in referring state patients back from court for admission and less than half of state patients were admitted within 6 months after observation despite there being no waiting list for admission of state patients in KwaZulu-Natal. This may be related to the lengthy legal proceedings especially in the case of re-referral for observation or request for the psychiatrist to give expert evidence in court. Another contributing factor may be the involvement of other stakeholders in this process such as the South African Police Services, National Department of Health and the Correctional Services Department. This again raises the issue of accused spending longer time in detention with its ethical implications.

The rate of re-referral for a second observation was consistent with the literature, Marais et al. reported similar results with 15% of patients requiring a repeat psychiatric observation.^4,6^ This revolving door phenomenon for a subgroup of accused re-sent for observation may be because of the unsatisfactory observation reports disputed by the court or the defence, request for another psychiatrist or psychologist, changes in the mental condition of the accused and additional information brought to court. In this study, accused have been re-referred for observation after more than a year in many cases making retrospective assessment of the mental state at the time of the crime very challenging.

When exploring agreement with the diagnosis between assessing and treating psychiatrists, there was a correlation with regard to the diagnosis of intellectual disability. Intellectual disability was the most common diagnosis on admission as state patient^5^ and in the observation report and this is supported by the literature. Yap et al. reported that offenders found unsound of mind were most commonly diagnosed with intellectual disability or schizophrenia.^6^ The finding that there was no diagnosis of mental illness in five (5.49%) cases in this study is similar to the finding by Yap et al. who also reported that 13.9% of observation reports had no mental illness diagnosed with a variation rate in the literature from 8% to 17% in other studies.^6^ This may be because of courts referring the accused for observation based on the presence of odd behaviour or when there is a serious charge, but not all serious charges or inappropriate behaviour is related to mental illness.

A considerable number of state patients denied committing the crime. The vulnerability of mentally ill patients is a major ethical concern in our practice because they can be unfit to stand trial but with a questionable involvement in the crime. They can falsely confess, and their credibility is also questioned as they can be suggestable.^9^ Accused is also generally referred for observation at a pre-trial stage when investigations for the offence are still in progress in most cases.

The finding that the majority of state patients were found to be not fit to stand trial is consistent with another local study.^4^ However, few state patients were found to be not criminally responsible, and this is not supported by local and international literature.^4,10,11^ The comparison of this result with international literature is to be considered with caution as legislations related to mentally ill offenders vary worldwide. Skipworth et al. reported that international comparison is complicated by the fact that forensic populations are usually defined legally, and with no two jurisdictions identical generalisation of findings is problematic.^12^ In this study, the finding is probably related to the high number of ‘no comments’ in the observation report about the criminal responsibility (section 78). This conclusion may confuse the court and impact its decision. Different factors may cause non-committal conclusions on criminal responsibility in the observation report by the assessing psychiatrist: firstly, a personal attitude of being reluctant to comment when the accused have repudiated the crime, and secondly, inadequate information and inconclusive diagnoses may lead to inconclusive reports. Delayed referral or insufficient information and finally experts not providing a joint consensus report as a panel may also contribute to the finding. This last theory is supported by a US study where the panel members do not communicate and each provides an independent report; the author found discrepancies between the reports and high percentage of no opinion and split decisions in the criminal responsibility group.^13^ Sharing opinions and discussing cases as a panel may allow for a more objective and accurate assessment of the criminal responsibility. Finally non-committal observation reports may be related to the court mixing both enquiries at the same time in all cases, despite the CPA, allowing the accused to be probably referred for observation in terms of section 77 or 78. These two enquiries are different especially with regard to the legal outcome, and it would be relevant to separate them as in other countries.^13,14^ This may be recommended for many reasons; in some cases the enquiry into the criminal responsibility (section 78) is not relevant leading to a non-conclusive report. This may have been prevented if the enquiry was more specific and only made in terms of section 77, especially when a long time has elapsed between the commission of the alleged offence and the enquiry. This may limit the ability of the psychiatrist to comment on the criminal responsibility.

Accused are referred for observation at a pre-trial stage, even before evidence against the accused is established. However, regarding the criminal responsibility, an indisputable aspect is that the accused must have committed the index offence, as it is nonsensical to plead lack of criminal capacity for a crime one did not commit.^15^

This is a major ethical issue that forensic psychiatrists are facing when dealing with observation cases. The enquiry may be made at a pre-trial stage, but it would be more relevant to enquire about the criminal responsibility at a trial stage.

Furthermore, accused found not fit to stand trial may at any time thereafter, when he or she is capable of understanding the proceedings, be prosecuted and tried for the offence in question.^2^ However, accused found not criminally responsible are declared not guilty. Although accused are declared state patients in both cases, the legal outcomes are different for the two sections. The enquiries under sections 77 and 78 are separate issues, and enquiring both of them at the same time may be confusing in some cases. Instead of making the enquiry in terms of both sections for all accused referred for observation, it would be recommended to address the enquiry in a single observation or separately depending on the case.

Some individuals were declared state patients even though they were not diagnosed with any mental illness; or they were found to be fit to stand trial and/or able to appreciate the wrongfulness of their acts; or when the assessing psychiatrist was not able to comment on the criminal responsibility. This reflects possible discrepancies between the clinician’s finding and the court’s decision. These findings are consistent with the literature.^5,13,14^ Discrepancies may be because of the absence of consensus or the presence of split opinions, or judges utilising other information concerning defendants not available to examiners.^14^ There were also cases where individuals were admitted as state patients for an indefinite period when they did not commit a serious offence and this is not in keeping with the provisions of the CPA.^2^ This raises again ethical questions as it may have considerable implications on the patient’s rights and could lead to stigmatisation of mentally ill patients who would be then considered dangerous by the society.

## Study limitations

Although the study provided substantially important findings, the generalisation of the study is limited as it was based at one institution, relied on retrospective chart records and was cross-sectional in nature. A prospective, longitudinal study of all observation cases and their outcomes in limited-resource settings is required.

## Conclusion and recommendations

The delay in referral for observation and for admission as a state patient; requests for re-observation; lack of consistency in reporting on criminal capacity; and detention of patients found to be both fit to stand trial and criminally responsible, or not to have a mental illness or who have not committed a serious crime, all serve to raise concern on forensic psychiatric practice. The study highlights the need for forensic psychiatry training updates, greater collaboration between assessing forensic psychiatrists and between the mental health and the justice systems to best utilise the limited resources and protect patient rights.
